# Supplementary material for: Retinal orientation and interactions in rhodopsin reveal a two-stage trigger mechanism for activation
Source: Nat Commun. 2016 Sep 2;7:12683. doi: 10.1038/ncomms12683 (PMC5025775; doi:10.1038/ncomms12683)
Supplement: Supplementary Information — Supplementary Figures 1-8, Supplementary Tables 1-6, Supplementary Notes 1-7, Supplementary References [file ncomms12683-s1.pdf]

Supplementary Figure 1

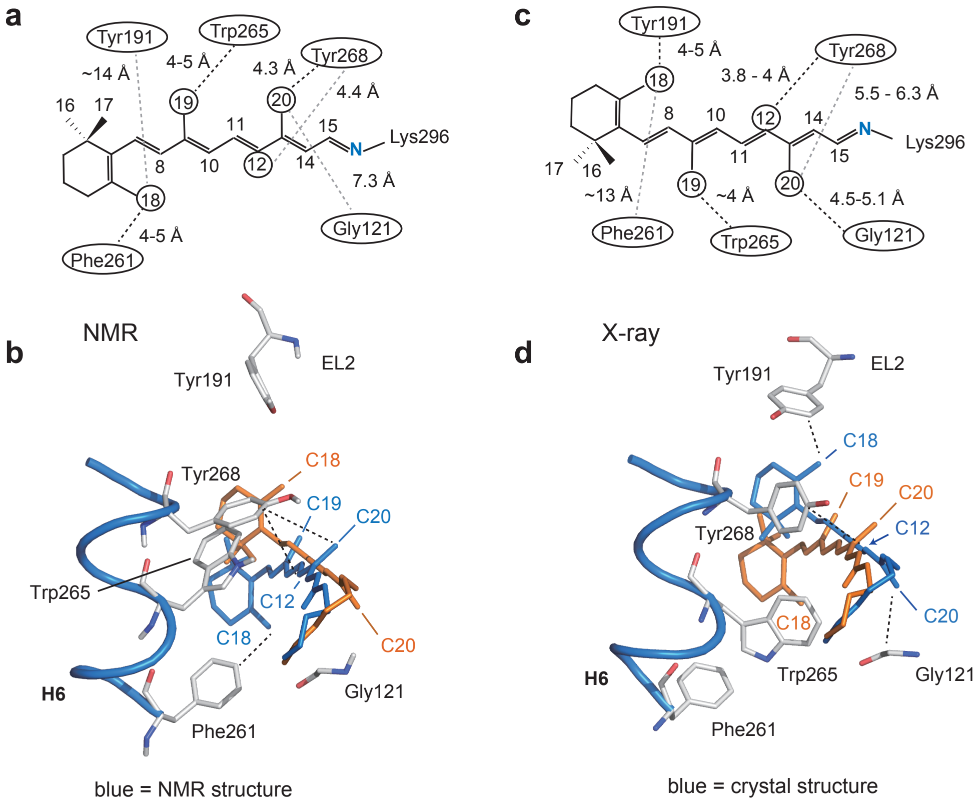

Supplementary Figure 1. Comparison of retinal orientations in Meta II studied by NMR and crystallography. (**a,c**) Schematics showing retinal-protein distances in Meta II defined by NMR (**a**) or crystallography (**c**). Internuclear distances between the C18, C19 and C20 methyl groups and several amino acids in the retinal binding site were taken from NMR-constrained MD simulations of Meta II[1] (**b**) or from the crystal structures of Meta II[4,5] (see Supplementary Tables 1-5) (**d**). The position of the retinal from the crystal structure of Meta-II-opsin (PDB ID 3PQR)[2] (**b**) and from the NMR-constrained MD simulations (**d**) is shown in orange for comparison. Dashed lines in (**b**) and (**d**) indicate close contacts between the retinal and specific binding site residues highlighted in (**a**) and (**c**), respectively.

Supplementary Figure 2

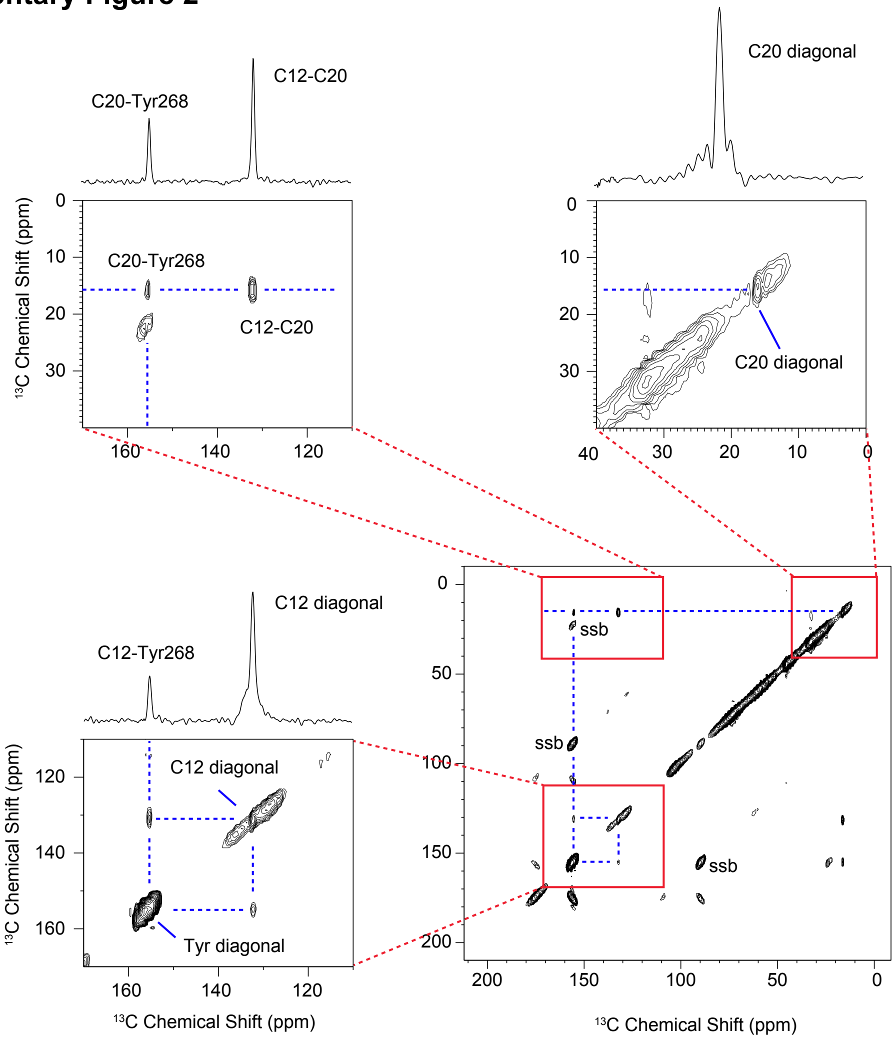

Supplementary Figure 2. Two-dimensional solid-state NMR of rhodopsin. In magic angle spinning NMR studies of GPCRs, structural information is obtained through measurements of chemical shifts and dipolar couplings[3,4]. The full 2D ^{13}C…^{13}C correlation NMR spectrum of rhodopsin labeled with ^{13}Cζ-tyrosine and regenerated with ^{13}C12, ^{13}C20 retinal is shown in the lower right panel as a contour plot. The NMR resonances emerge from the plane of the paper and their intensities are best characterized by taking one-dimensional rows through the spectrum. The resonances along the diagonal of the 2D plot correspond to the 1D NMR spectrum. Off-diagonal crosspeaks arise from ^{13}C sites that are separated in space by less than ~6 Å. Crosspeak intensity is inversely related to internuclear distance. The strongest off-diagonal crosspeaks are observed between the retinal ^{13}C12 and ^{13}C20 resonances (upper left panel). A fixed distance (2.4 Å) separates these ^{13}C sites in the retinal structure. The intensity of the ^{13}C12-^{13}C20 "internal control" allows us to scale the ^{13}C12-^{13}Cζ-Tyr268$^{6.51}$ and the ^{13}C20-

^{13}Cζ-Tyr268$^{6.51}$ crosspeaks relative to each other. Strong crosspeaks are observed between the ^{13}C20 retinal resonance and the ^{13}Cζ-resonance of Tyr268$^{6.51}$ (upper left panel). The weaker crosspeak between the ^{13}C12 and ^{13}Cζ-Tyr268$^{6.51}$ resonances (relative to the ^{13}C12-^{13}C20 "internal control") indicates a longer internuclear distance (lower left panel). Spinning side bands (ssb) generated by magic angle spinning can produce artifacts in the 2D spectrum. These are labeled as "*ssb*".

Supplementary Figure 3

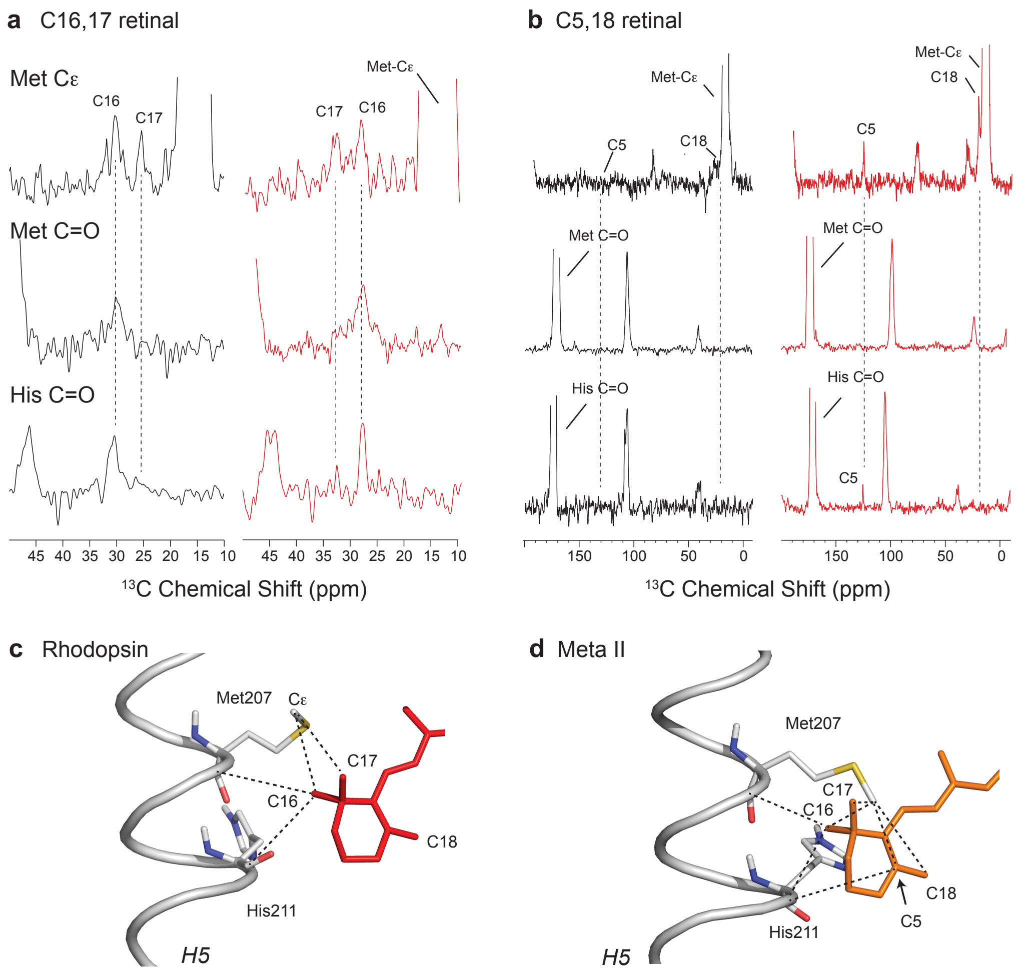

Supplementary Figure 3. NMR constraints on the position of the β-ionone ring in Meta-II. (**a, b**) ^{13}C DARR NMR measurements are shown of rhodopsin labeled with ^{13}Cε-Met, ^{13}C=O Met or ^{13}C=O His and regenerated with ^{13}C16, ^{13}C17 11-*cis* retinal (**a**) or with ^{13}C5, ^{13}C18-labeled 11-*cis* retinal (**b**). Selected rows from the DARR experiment through the ^{13}Cε-Met, ^{13}C=O Met or ^{13}C=O diagonal resonances are shown for rhodopsin (black) and Meta-II (red). (**a**) In rhodopsin, the resonances at 30.6 and 26.1 ppm are assigned to the ^{13}C16 and ^{13}C17 carbons, respectively[5]. In solution, the ^{13}C16 and ^{13}C17 resonances are at the same chemical shift of ~28.9 ppm in both 11-*cis* PSB and all-*trans* SB retinal model compounds[6]. (**c,d**) Position of the β-ionone ring relative to Met207$^{5.42}$ and His211$^{5.46}$ in the rhodopsin crystal structure (PDB ID 1U19) (**c**) and Meta-II structure (**d**) taken from guided-MD simulations[1]. Internuclear ^{13}C...^{13}C distances constraints on the position of the ring are shown as dashed lines.

Supplementary Figure 4

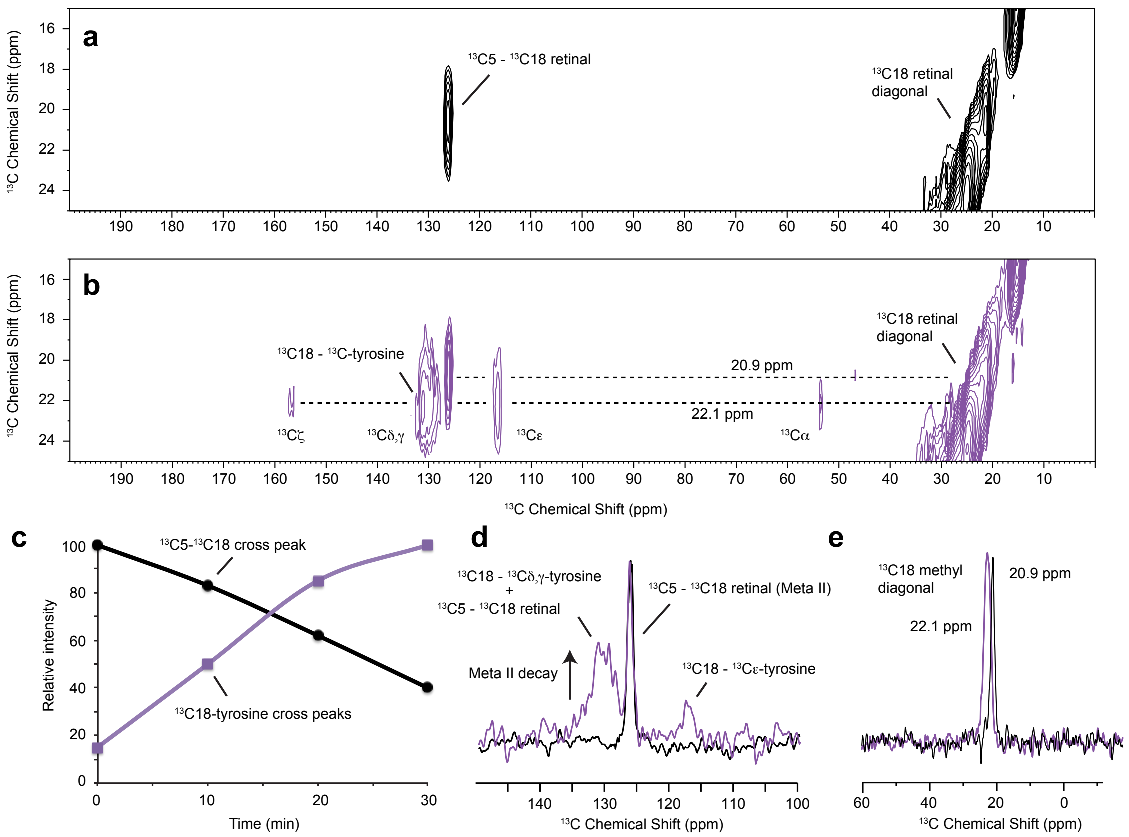

Supplementary Figure 4. Reorientation of the β-ionone ring upon decay of Meta-II. The region of the 2D DARR NMR spectrum corresponding to the ^{13}C5-^{13}C18 crosspeaks and ^{13}C18-tyrosine crosspeaks is shown in panel (**a**) for Meta-II and in panel (**b**) during the decay of Meta-II to opsin. In Meta-II (**a**), crosspeaks between the retinal ^{13}C18 methyl resonance and ^{13}C-tyrosine resonances are not observed. As Meta-II decays, we begin to observe resonances that can be attributed to crosspeaks between the ^{13}C18 retinal methyl group and tyrosine, which increase in intensity after each warm-cool cycle (**c**, purple filled squares). The spectrum in panel (**b**) was obtained after three warm-cool cycles. Resonances at 53 ppm, 117 ppm, ~130 ppm and 157 ppm originating from U-^{13}C-tyrosine are clearly observed. Panel (**d**) shows the row through the C18 diagonal in Meta II showing the intense C5-C18 crosspeak (black trace). There is very little intensity to tyrosine resonances. Comparison of the same row after the decay of Meta II (purple trace) shows a strong increase in the C18-tyrosine crosspeaks. The spectra are normalized to the C5-C18 crosspeak to emphasize the increase in the C18-Tyr crosspeak relative to the C5-C18 crosspeak as Meta II decays. (**e**) Rows taken through the diagonal ^{13}C18 methyl resonances at 20.9 ppm (black trace) and 22.1 ppm (purple trace).

Supplementary Figure 5

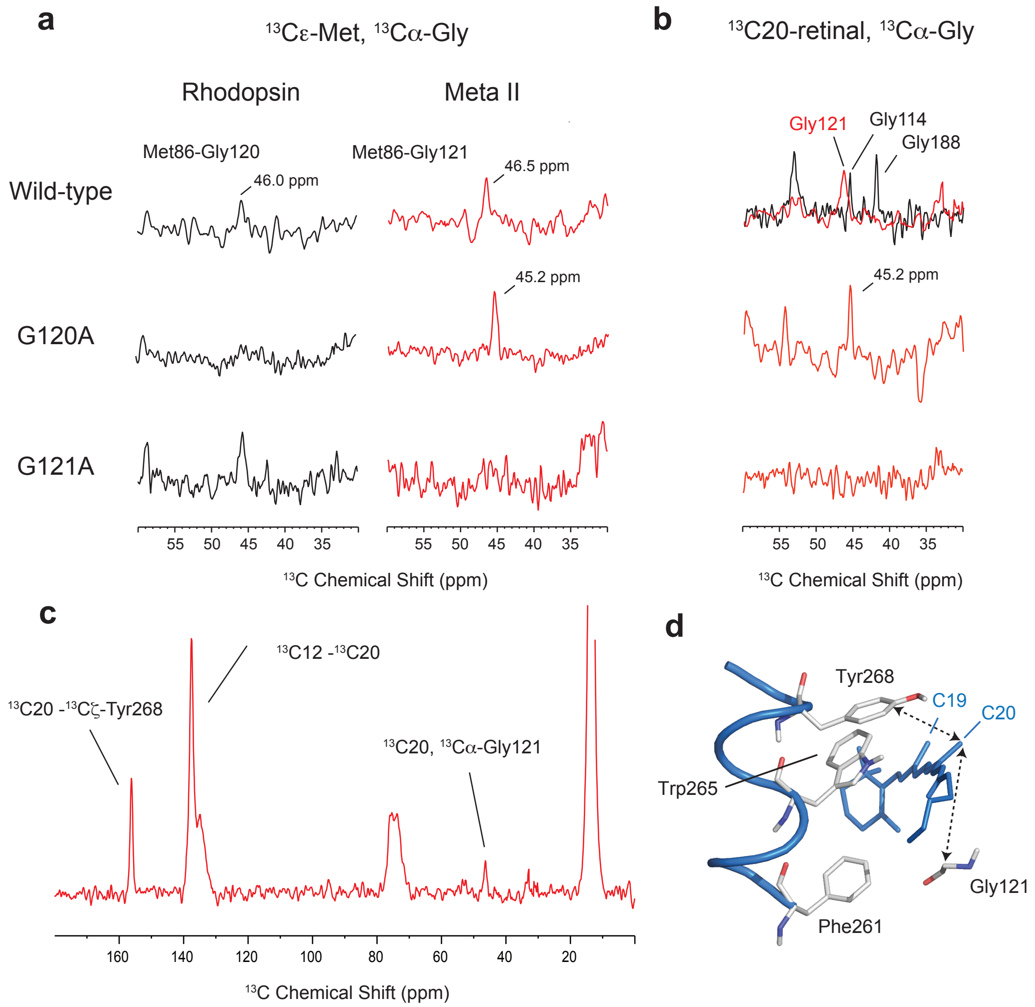

Supplementary Figure 5. Assignment of the tyrosine-glycine crosspeak in Meta-II. (**a**) 2D DARR NMR experiments on ^{13}Cε-Met, ^{13}Cα-Gly – labeled rhodopsin are shown to determine the frequency of the ^{13}Cα chemical shifts of Gly120$^{3.35}$ and Gly121$^{3.36}$. Rows are taken through the Met86$^{2.53}$ diagonal resonance at 13.6 ppm in rhodopsin (black) and 15.0 ppm in Meta II (red). There is a single crosspeak assigned to Met86$^{2.53}$-Gly120$^{3.35}$, the only Met-Gly pair within 6 Å in rhodopsin (black spectrum, first column)[7]. The resonance is present in the G121A mutant (with a slightly lower chemical shift of 45.2 ppm), but absent in the G120A mutant. In Meta II, a Met-Gly crosspeak is observed at 46.5 ppm. This crosspeak is assigned to Gly121$^{3.36}$ rather than Gly120$^{3.35}$ on the basis that this Met-Gly crosspeak is lost in the G121A mutant, but maintained in the G120A mutant at a lower chemical shift of 45.2 ppm. This observation indicates that Met86$^{2.53}$ shifts from contacting Gly120$^{3.35}$ in rhodopsin to contacting Gly121$^{3.36}$ in Meta II. (**b**)

^{13}C DARR measurements of wild-type, G120A and G121A rhodopsin labeled with ^{13}Cα-glycine and containing ^{13}C12, ^{13}C20 retinal. In rhodopsin (top panel, black), there are two resonances observed that can be assigned to Gly114$^{3.29}$ and Gly188^{EL2}. Upon conversion to Meta-II (top panel, red), there is a single crosspeak at ~46.3 ppm that is assigned to Gly121$^{3.36}$ on the basis of the G120A and G121A mutants. In (**b**), the rows are taken through the ^{13}C20 diagonal resonance at 16.4 ppm in rhodopsin and 13.7 ppm in Meta II. These data confirm the assignment of the ^{13}C20-Gly crosspeak to Gly121$^{3.36}$. (**c**) Row through the retinal ^{13}C20 resonance at 13.7 ppm of a DARR spectrum of Meta-II labeled with ^{13}Cζ-tyrosine, ^{13}Cα-glycine and containing ^{13}C12, ^{13}C20 retinal. The crosspeaks of the ^{13}C20 methyl group to the ^{13}C12 retinal, Tyr268$^{6.51}$ and Gly121$^{3.36}$ resonances are marked. (**d**) Orientation of the retinal chromophore in Meta II based on NMR-constrained MD simulations. The distances between the retinal C20 methyl group and Gly121$^{3.36}$ and Tyr268$^{6.51}$ are constrained by the relative intensities of the crosspeaks observed in panel (**c**). A rotation of about the long axis of the retinal to bring the C20 methyl group closer to Gly121$^{3.36}$ would increase the separation with Tyr268$^{6.51}$, which is not observed.

Supplementary Figure 6

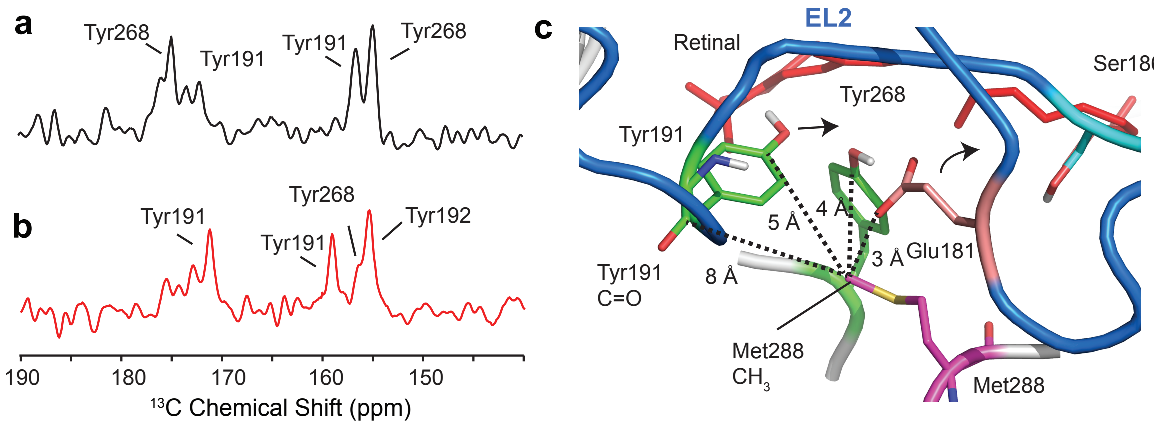

Supplementary Figure 6. Interaction of Met288$^{7.35}$ with Tyr191^{EL2}. **(a)** 2D ^{13}C DARR NMR of rhodopsin (**a**) and Meta-II (**b**) containing ^{13}Cε-methionine and U-^{13}C-tyrosine. The row is shown through the diagonal resonance of ^{13}Cε-Met288$^{7.35}$ at 17.2 ppm (**a**) and 12.2 ppm (**b**). In rhodopsin, crosspeaks are observed between the ^{13}Cε-Met288$^{7.35}$ diagonal resonance and both the Tyr268$^{6.51}$ ^{13}Cζ and ^{13}C=O resonances at 156.3 and ~175 ppm, respectively. In Meta II, strong crosspeaks are observed between the ^{13}Cε-Met288$^{7.35}$ diagonal resonance and both the Tyr191^{EL2} ^{13}Cζ and ^{13}C=O resonances at 159.3 and ~172 ppm, respectively. (**c**) Extracellular view of residues in the region of the retinal protonated Schiff base in the crystal structure of rhodopsin (PDB ID 1U19). Arrows indicate the proposed motion of Glu181^{EL2} in the transition to Meta-I and of Tyr191^{EL2} in the transition to Meta-II.

Supplementary Figure 7

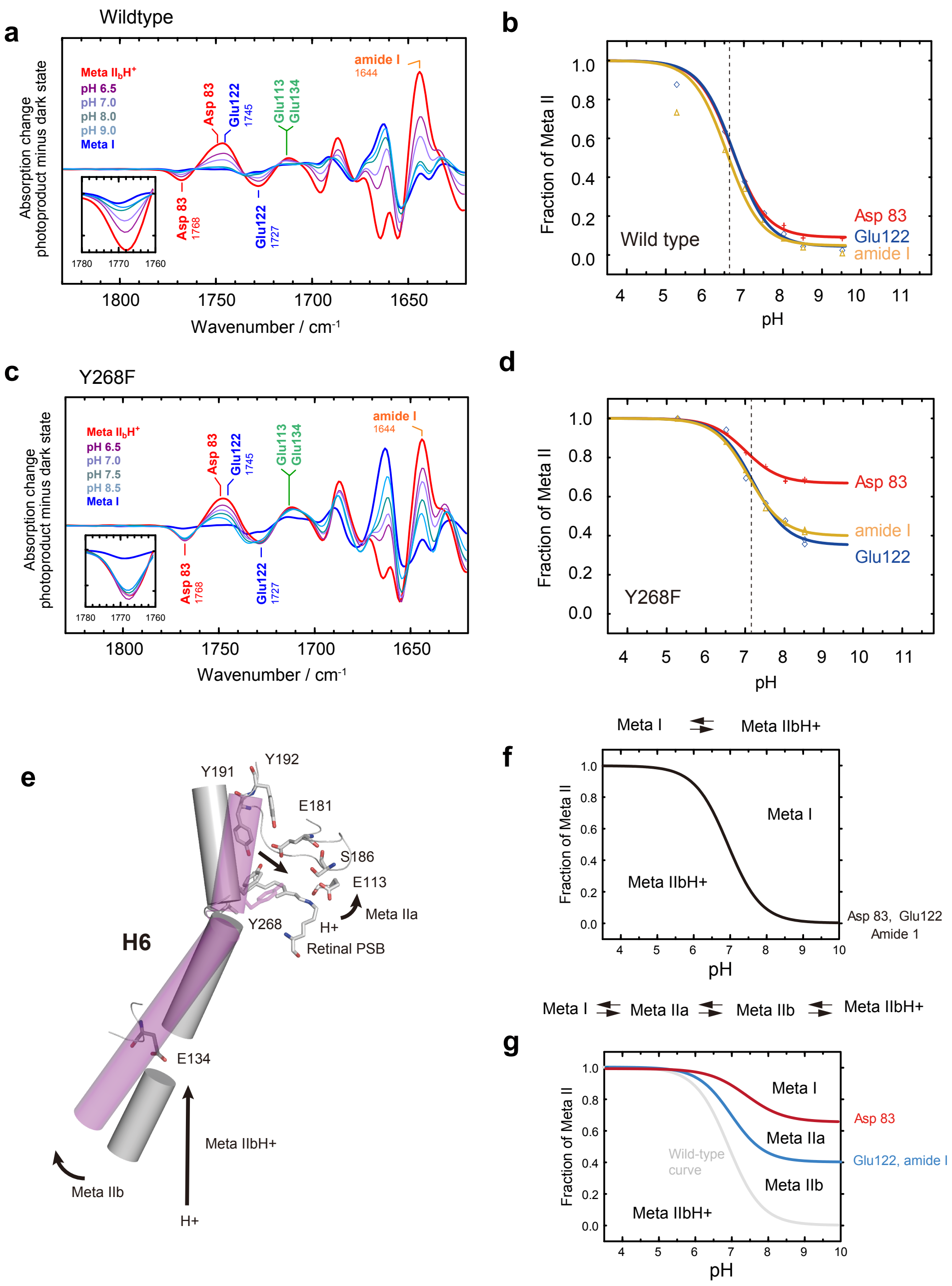

Supplementary Figure 7: FTIR analysis of rhodopsin mutants. (**a**) Difference FTIR spectra of wild-type rhodopsin (**a**) and the Y268F mutant (**c**) between 1620-1850 cm^{-1} are shown for Meta-I (blue) and Meta-IIbH+ (red) minus rhodopsin. Plots derived from these spectra present the fraction of Meta-II as a function of pH for wildtype (**b**) and the Y268F mutant (**d**). For the wildtype receptor, each of the three signature bands titrates in unison with a well-defined pKa

reflecting a two state transition. In the case of Y268F, there is a dramatic difference in the titration behavior of the Asp83$^{2.50}$ vibration when compared to the signature bands for Glu122$^{3.37}$ or the amide I vibration, indicating that mutations (such as Y268F) can result in the stabilization of additional Meta-II substates. (**e**) Cartoon of H6 in rhodopsin illustrating the different substates occurring between Meta I and Meta IIbH+. The purple cylinders are a cartoon representation of the mechanism proposed here in which deprotonation and the associated changes in the extracellular hydrogen-bonding network allow the intracellular end of H6 to pivot inward and the extracellular end of H6 to pivot outward. (**f**) At low temperature (e.g. 0 °C for the FTIR measurements reported here), the steps involved in Meta-II formation occur synchronously and a two-state transition between Meta-I and Meta-IIbH+ is observed. (**g**) At higher temperatures or in the case of several specific mutations at 0 °C (e.g. Y191F and Y268F), the three FTIR vibrations used to probe the Meta-I to Meta-II transition titrate with different pKa values indicating additional substates[8]. At higher pH values, an equilibrium exists between Meta-I, Meta-IIa and Meta-IIb. As seen for the Y268F mutant in panel (**c**), the Asp83 titrates rapidly indicating a strong forward shift in the equilibrium in this mutant.

Supplementary Figure 8

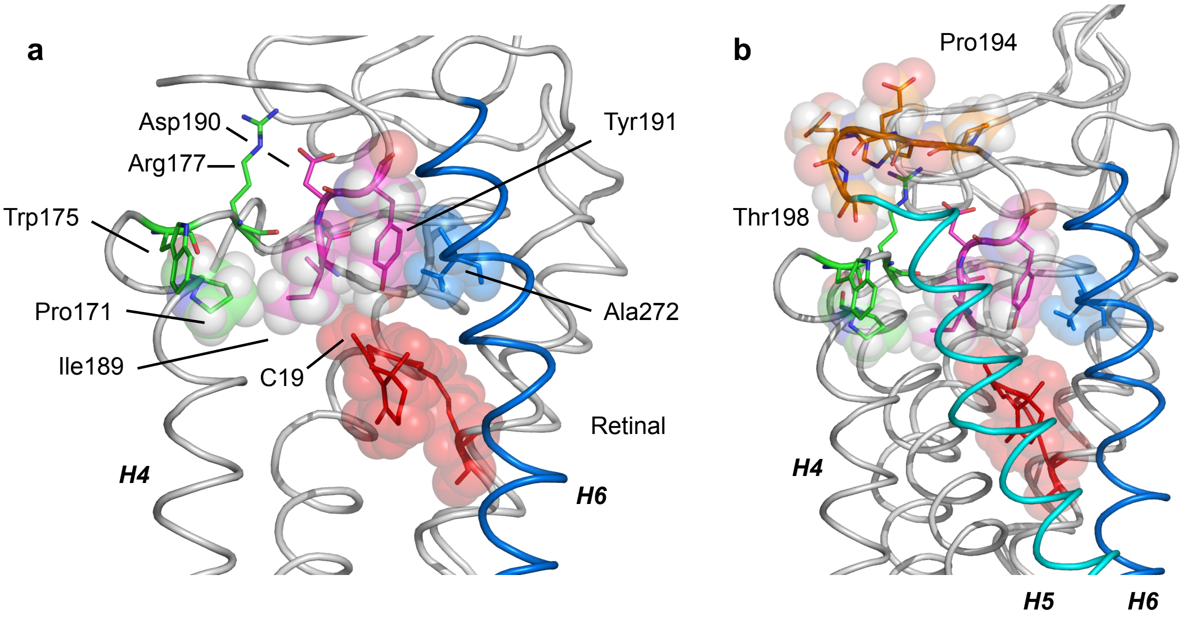

Supplementary Figure 8: Conserved residues involved in the Tyr191^{EL2} trigger. Tyr191^{EL2} and Tyr268$^{6.51}$ are key aromatic residues on the extracellular side of rhodopsin that contribute to the second stage of the proposed two-stage trigger. (**a**) Tyr191^{EL2} is packed against the retinal C19 methyl group of the retinal, Ala272 $^{6.55}$ on TM helix H6 and Ile189 ^{EL2} on EL2. Ile189^{EL2} is also packed against Pro171$^{4.60}$, which is one of the most highly conserved residues in the visual receptors (96%) and whose C=O hydrogen bonds to the backbone NH of Arg177^{EL2}. Arg177^{EL2} forms a conserved salt bridge with Asp190^{EL2} that is involved in receptor stability[9]. Pro171$^{4.60}$ packs against Ser176^{EL2}, which in turn packs against Trp175^{EL2} whose mutation leads to constitutive activity[10]. (**b**) A second cluster of amino acids between Tyr191^{EL2} and the extracellular end of H5 (Pro194^{EL2}-Thr198^{EL2}) are of interest because they are *not* conserved between the bovine and human rhodopsin. Overall, bovine and human rhodopsin have a sequence identity (93.4%), and this is one of the few regions that is not conserved. Kazmin *et al.*[11] using receptor chimera have found that this region influences the coupling between EL2 and H5 and rate of Meta-II decay. They show by FTIR spectroscopy that significant differences between bovine and human rhodopsin already occur in the formation of Meta-I, which suggests that the extracellular region of rhodopsin already has started to rearrange before the transition to the active Meta-II state.

Supplementary Table 1. Retinal (C5, C16-C18) - Protein Distances in Rhodopsin and Meta-II

		1U19 Rho	1GZM Rho	3PXO Meta-II	3PQR Meta-II	4A4M Meta-II	Guided MD
C5	^{13}Cε-Met207	6.70 Å	6.72 Å	4.24 Å	3.84 Å	4.40 Å	5.37 Å
C16	^{13}Cε-Met207	3.79 Å	3.47 Å	5.63 Å	5.65 Å	5.03 Å	3.63 Å
C17	^{13}Cε-Met207	4.53 Å	4.34 Å	3.66 Å	3.77 Å	3.49 Å	4.78 Å
C18	^{13}Cε-Met207	7.71 Å	7.84 Å	4.24 Å	4.35 Å	4.67 Å	5.56 Å
C5	^{13}C=O-Met207	8.21 Å	7.85 Å	5.52 Å	5.43 Å	5.42 Å	7.63 Å
C16	^{13}C=O-Met207	4.90 Å	4.71 Å	6.33 Å	6.24 Å	6.34 Å	4.49 Å
C17	^{13}C=O-Met207	6.98 Å	6.88 Å	4.32 Å	4.25 Å	3.86 Å	6.23 Å
C18	^{13}C=O-Met207	9.26 Å	8.97 Å	6.26 Å	6.24 Å	6.24 Å	8.69 Å
C5	^{13}C=O-His211	6.74 Å	6.17 Å	7.19 Å	7.15 Å	7.18 Å	5.42 Å
C16	^{13}C=O-His211	4.87 Å	4.94 Å	5.53 Å	5.51 Å	5.22 Å	4.18 Å
C17	^{13}C=O-His211	7.15 Å	7.02 Å	3.84 Å	3.73 Å	3.93 Å	6.45 Å
C18	^{13}C=O-His211	7.61 Å	7.11 Å	8.30 Å	8.34 Å	8.40 Å	6.33 Å
C18	^{13}Cζ-Tyr191	9.31 Å	8.95 Å	5.82 Å	5.47 Å	5.26 Å	14.4 Å
C18	^{13}Cζ-Phe261	4.92 Å	5.06 Å	12.39 Å	12.3 Å	12.7 Å	4.01 Å
C18	^{13}Cε2-Phe261	5.90 Å	5.28 Å	12.64 Å	12.7 Å	13.2 Å	4.14 Å
C18	^{13}Cε1-Phe261	5.38 Å	6.19 Å	13.09 Å	13.0 Å	13.1 Å	5.13 Å
C18	^{13}Cα-Gly121	3.71 Å	3.56 Å	10.85 Å	10.3 Å	11.7 Å	5.64 Å
C18	^{13}C=O-Gly121	3.76 Å	3.66 Å	10.41 Å	10.7 Å	11.2 Å	4.60 Å

Supplementary Table 2: Retinal (C8,C19) - Protein Distances in Rhodopsin and Meta-II

		**1U19 Rho**	**1GZM Rho**	**3PXO Meta-II**	**3PQR Meta-II**	**4A4M Meta-II**	**Guided MD**
C8	^{13}Cζ-Tyr268	4.42 Å	4.60 Å	4.78 Å	4.93 Å	4.61 Å	7.51 Å
C8	^{13}Cζ- Phe261	7.95 Å	7.91 Å	10.47 Å	10.4 Å	11.43 Å	5.27 Å
C8	^{13}Cε2- Phe261	8.69 Å	8.33 Å	10.89 Å	10.9 Å	11.61 Å	5.46 Å
C19	^{13}Cε1- Phe261	8.53 Å	11.23 Å	10.47 Å	10.7 Å	10.53 Å	6.49 Å
C19	^{13}Cζ-Tyr268	4.32 Å	4.81 Å	6.25 Å	6.36 Å	5.54 Å	6.39 Å
C19	^{13}Cζ-Tyr191	4.69	4.58 Å	9.34 Å	9.19 Å	8.81 Å	9.48 Å
C19	^{13}C=O-Gly121	8.71 Å	8.50 Å	5.30 Å	5.58 Å	6.12 Å	7.24 Å
C19	^{13}Cη2-Trp265	6.71 Å	6.49 Å	4.25 Å	4.63 Å	4.06 Å	3.94 Å
C19	^{13}Cζ3-Trp265	6.10 Å	5.97 Å	3.90 Å	4.25 Å	3.67 Å	4.99 Å

Supplementary Table 3. Retinal (C12, C20) - Protein Distances in Rhodopsin and Meta-II

		1U19 Rho	1GZM Rho	3PXO Meta-II	3PQR Meta-II	4A4M Meta-II	Guided MD
C12	$^{13}C\alpha$-Gly114	4.19 Å	4.78 Å	8.12 Å	7.99 Å	7.49Å	6.02 Å
C12	$^{13}C\alpha$-Gly121	8.36 Å	8.41 Å	6.15 Å	6.06 Å	6.94 Å	6.35 Å
C12	$^{13}C\alpha$-Gly188	4.28 Å	3.92 Å	6.05 Å	5.98 Å	5.23 Å	6.93 Å
C12	$^{13}C\zeta$-Tyr268	4.91 Å	4.45 Å	3.81 Å	3.87 Å	4.01 Å	4.44 Å
C20	$^{13}C\alpha$-Gly114	7.16 Å	7.23 Å	7.51 Å	7.41 Å	8.34 Å	4.7 Å
C20	$^{13}C\alpha$-Gly120	9.43 Å	9.40 Å	6.31 Å	6.25 Å	7.35 Å	8.45 Å
C20	$^{13}C\alpha$-Gly121	7.95 Å	7.82 Å	4.75 Å	4.48 Å	5.14 Å	7.3 Å
C20	$^{13}C\alpha$-Gly188	6.16 Å	6.05 Å	7.07 Å	7.10 Å	7.41 Å	5.8 Å
C20	$^{13}C\alpha$-Thr118	6.64 Å	6.59 Å	4.21 Å	4.08 Å	5.14 Å	4.00 Å
C20	^{13}C=O-Thr118	7.90 Å	7.81 Å	5.09 Å	4.96 Å	6.00 Å	5.52 Å
C20	$^{13}C\zeta$-Tyr268	4.18 Å	4.10 Å	6.24 Å	6.30 Å	5.52 Å	4.28 Å

Supplementary Table 4. Retinal (C14, C15) - Protein Distances in Rhodopsin and Meta-II

		1U19 Rho	1GZM Rho	3PXO Meta-II	3PQR Meta-II	4A4M Meta-II	Guided MD
C14	^{13}C=O-Cys185	7.60 Å	7.50 Å	9.96 Å	9.98 Å	9.63 Å	12.40 Å
C14	^{13}Cα-Cys185	9.00 Å	8.90 Å	11.13 Å	11.18 Å	10.86 Å	13.77 Å
C14	^{13}Cβ-Cys185	9.60 Å	9.63 Å	11.36 Å	11.52 Å	11.25 Å	14.55 Å
C14	^{13}C=O-Ser186	5.59 Å	5.32 Å	7.58 Å	7.64 Å	7.29 Å	9.68 Å
C14	^{13}Cα-Ser186	5.41 Å	5.27 Å	7.82 Å	7.85 Å	7.37 Å	10.03 Å
C14	^{13}Cβ-Ser186	4.49 Å	4.32 Å	6.63 Å	6.66 Å	6.13 Å	9.14 Å
C14	^{13}C=O-Cys187	4.97 Å	4.71 Å	6.44 Å	6.49 Å	6.17 Å	7.59 Å
C14	^{13}Cα-Cys187	5.60 Å	5.40 Å	7.58 Å	7.59 Å	7.16 Å	8.63 Å
C14	^{13}Cβ-Cys187	6.57 Å	6.45 Å	8.67 Å	8.67 Å	8.16 Å	8.92 Å
C14	^{13}Cζ-Tyr268	6.03 Å	5.67 Å	4.88 Å	5.00 Å	5.25 Å	4.54 Å
C15	^{13}C=O-Cys185	7.13 Å	7.18 Å	9.23 Å	9.29 Å	9.51 Å	11.05 Å
C15	^{13}Cα-Cys185	8.48 Å	8.56 Å	10.43 Å	10.54 Å	10.71 Å	12.44 Å
C15	^{13}Cβ-Cys185	8.87 Å	9.07 Å	10.56 Å	10.80 Å	10.92 Å	13.19 Å
C15	^{13}C=O-Ser186	5.75 Å	5.65 Å	7.21 Å	7.30 Å	7.62 Å	8.49 Å
C15	^{13}Cα-Ser186	5.20 Å	5.23 Å	7.19 Å	7.26 Å	7.41 Å	8.72 Å
C15	^{13}Cβ-Ser186	4.05 Å	4.00 Å	5.88 Å	5.97 Å	6.04 Å	7.80 Å
C15	^{13}C=O-Cys187	5.94 Å	5.85 Å	6.56 Å	6.63 Å	7.07 Å	6.77 Å
C15	^{13}Cα-Cys187	6.31 Å	6.30 Å	7.49 Å	7.54 Å	7.85 Å	7.66 Å
C15	^{13}Cβ-Cys187	7.34 Å	7.40 Å	8.53 Å	8.56 Å	8.83 Å	8.00 Å
C15	^{13}Cζ-Tyr268	6.68 Å	6.40 Å	6.18 Å	6.31 Å	6.12 Å	5.00 Å

Supplementary Table 5. Selected Internuclear Protein-Protein Distances in Rhodopsin and Meta-II

		1U19 Rho	1GZM Rho	3PXO Meta-II	3PQR Meta-II	4A4M Meta-II	Guided MD
$^{13}C\alpha$-Gly114	$^{13}C\zeta$-Tyr268	8.86 Å	8.74 Å	10.51 Å	10.59 Å	10.97 Å	8.07 Å
$^{13}C\alpha$-Gly114	$^{13}C\zeta$-Tyr178	4.48 Å	4.51 Å	4.53 Å	4.66 Å	4.48 Å	5.34 Å
$^{13}C\alpha$-Gly188	$^{13}C\zeta$-Tyr268	5.35 Å	4.99 Å	6.68 Å	6.79 Å	7.05 A	5.21 Å
$^{13}C\alpha$-Gly188	$^{13}C\zeta$-Tyr178	6.19 Å	6.47 Å	6.04 Å	6.24 Å	6.40 Å	7.97 Å
$^{13}C\varepsilon$-Met288	$^{13}C\zeta$-Tyr268	3.88 Å	3.63 Å	8.10 Å	8.23 Å	8.31 Å	3.76 Å
$^{13}C\varepsilon$-Met288	$^{13}C\zeta$-Tyr191	5.16 Å	4.95 Å	4.11 Å	4.21 Å	4.19 Å	3.75 Å
$^{13}C\varepsilon$-Met288	^{13}C=O-Tyr191	8.72 Å	8.15 Å	6.47 Å	6.53 Å	6.32 Å	8.74 Å
$^{13}C\beta$-Ser186	^{13}C=O-Cys185	3.47 Å	3.33 Å	3.36 Å	3.34 Å	3.50 Å	3.37 Å
$^{13}C\beta$-Ser186	^{13}C=O-Cys187	5.03 Å	5.07 Å	4.94 Å	4.99 Å	4.95 Å	5.07 Å
$^{13}C\beta$-Se298	^{13}C=O-Cys185	6.23 Å	6.17 Å	6.47 Å	6.36 Å	6.30 Å	5.85 Å
$^{13}C\beta$-Ser298	^{13}C=O-Cys264	6.36 Å	6.53 Å	6.27 Å	6.22 Å	6.41 Å	7.23 Å
HO-Tyr268 [1]	HO-Tyr191 [1]	2.47 Å	2.73 Å	5.40 Å	5.51 Å	5.48 Å	4.68 Å
HO-Tyr268 [2]	COO-Glu181 [2]	3.01 Å	2.68 Å	2.98 Å	3.33 Å	3.80 Å	6.72 Å

[1,2] The distances listed are the C-OH oxygen distances between tyrosines and the C-OH Tyr268 oxygen to the closest oxygen of the Glu181 side chain carboxyl group.

Supplementary Table 6: Primer pairs used to construct mutants.

Mutant [1]	DNA sequence of mutagenic primer pairs
G120A fwd:	gcttctttgccaccctggccggtgaaattgcac
G120A rvs	gtgcaatttcaccggccagggtggcaaagaagc
G121A fwd	ctttgccaccctgggcgcagaaattgcactgtg
G121A rvs:	cacagtgcaatttctgcgcccagggtggcaaag
W175F fwd	ccgccgctcgtcggcttctctagatacatcccg
W175F rvs	cgggatgtatctagagaagccgacgagcggcgg
G188A fwd	ggcatgcagtgctcgtgcgccatcgattactacacg
G188A rvs:	cgtgtagtaatcgatggcgcacgagcactgcatgcc
Y178F fwd:	gtcggctggtctagattcatcccggagggcatg
Y178F rvs:	catgccctccgggatgaatctagaccagccgac
Y191F fwd	ctcgtgcgggatcgatttctacacgccgcacgag
Y191F rvs	ctcgtgcggcgtgtagaaatcgatcccgcacgag
Y192F fwd	cgtgcgggatcgattacttcacgccgcacgaggag
Y192F rvs	ctcctcgtgcggcgtgaagtaatcgatcccgcacg
M288A fwd	gcccatcttcgccaccatcccggctttctttgccaag
M288A rvs	cttggcaaagaaagccgggatggtggcgaagatgggc
M288L fwd	gcccatcttcctgaccatcccggctttctttgccaag
M288L rvs	cttggcaaagaaagccgggatggtcaggaagatgggc
Y268F	See Nakayama T. and Khorana, H.G. (1991) J. Biol. Chem. 266, 4269. Mutant made in Khorana lab by fragment replacement.

[1]All primers, forward (fwd) and reverse (rvs) are shown in the 5’-3’ direction. Mutant codons are underlined. Mutants were prepared by Quikchange site directed mutagenesis (Stratagene) unless stated otherwise.

Supplementary Notes

Supplementary Note 1. Retinal orientations differ in Meta II studied by NMR and crystallography.

There are two major differences between the orientation of the all-*trans* retinal SB chromophore in Meta II determined on the basis of NMR distance constraints and defined by electron density in protein crystal structures. The largest difference is the orientation of the β-ionone ring. In the NMR structure, the C18 methyl group is oriented toward $Phe261^{6.44}$, while in the crystal structures the C18 methyl group is oriented toward $Tyr191^{EL2}$ (Supplementary Fig. 1). We find that the retinal reorients during the decay of Meta II to opsin. This change positions the C18 methyl group in close proximity with $Tyr191^{EL2}$ (Supplementary Fig. 4, Supplementary Note 3).

The second, more subtle difference, is in the orientation of the retinal C19 and C20 methyl groups on the retinal polyene chain. These methyl groups have an orientation toward the extracellular surface in the NMR structure (as in the bathorhodopsin[12] and lumirhodopsin[13] crystal structures) and toward the cytoplasmic surface In the Meta-II crystal structures (Supplementary Fig. 1).

The position of C20 is constrained by intense NMR crosspeaks observed with the ^{13}Cζ-resonance of $Tyr268^{6.51}$ and by a weaker crosspeak to $Gly121^{3.36}$ (Supplementary Fig. 5). The C12 and C20 carbons are approximately equidistant from Cζ- $Tyr268^{6.51}$ (see Fig. 1 main text). In contrast, the relative orientations of the retinal and $Tyr268^{6.51}$ in the Meta II crystal structures place C12 much closer to Cζ- $Tyr268^{6.51}$ than C20. We propose that there is a slight inward tilt of TM helix H6 in order to satisfy the NMR constraints of the C20 methyl group to both $Gly121^{3.36}$ and $Tyr268^{6.51}$. In our previous studies, the guided MD simulations were undertaken using rhodopsin (PDB ID 1U19) as the starting structure and consequently did not incorporate the large outward rotation of the intracellular end of TM helix H6. Our current studies (see Supplementary Fig. 5) indicate that the C20 methyl group is closer to $Gly121^{3.36}$ than shown in Supplementary Fig. 1b, *i.e.* intermediate between the two structures shown above.

Supplementary Note 2. NMR constraints on the position of the β-ionone ring in Meta-II.

The position of the β-ionone ring differs in the structures determined using MD simulations guided by ^{13}C-solid-state NMR experiments[1], deuterium NMR experiments[14] and protein crystallography. In Supplementary Fig. 3, we present several additional solid-state NMR experiments that constrain the position of the β-ionone ring in Meta-II. In these experiments,

rhodopsin was labeled with either ^{13}Cε-methionine, ^{13}C=O methionine or ^{13}C=O histidine, and regenerated with either ^{13}C16, ^{13}C17 11-*cis* retinal or ^{13}C5, ^{13}C18-labeled 11-*cis* retinal.

For the experiments with ^{13}C16, ^{13}C17 11-*cis* retinal, the difference in chemical shift between the ^{13}C16 and ^{13}C17 resonances for retinal in solution and bound to rhodopsin is attributed to the inability of different ring conformations to rapidly interconvert within the receptor binding pocket[5]. Crosspeaks are observed between the ^{13}C16 resonance and the ^{13}C=O resonances of both Met207$^{5.42}$ and His211$^{5.46}$ (Supplementary Fig. 3) consistent with their distances in the crystal structure (4.9 Å, PDB ID 1U19). In the conversion to Meta-II, the upfield resonance now exhibits crosspeaks to the C=O of Met207$^{5.42}$ and His211$^{5.46}$. We have proposed that this change is due to ring inversion and a change in the orientation of the methyl groups (axial to equatorial) in the conversion to Meta-II rather than rotation about the C6-C7 single bond[5]. We believe that the ring is still in a constrained environment and not able to rapidly flip between different conformations in Meta-II since a mixture of axial – equatorial orientations or a mixture of rotamers about the C6-C7 bond would result in broadened lines at an averaged chemical shift.

For the experiments with ^{13}C5, ^{13}C18-labeled 11-*cis* retinal, these retinal positions are outside of the distance range of the DARR NMR experiment to Met207$^{5.42}$ and His211$^{5.46}$ in rhodopsin and consequently crosspeaks are not observed (Supplementary Fig. 3). In the conversion to Meta-II, crosspeaks are observed between both the ^{13}C5 and ^{13}C18 retinal resonances and ^{13}Cε-Met207$^{5.42}$. The observation of these crosspeaks is consistent with the position of the β-ionone ring in the Meta-II crystal structures and the structure based on MD simulations (Supplementary Table 1). However, the crosspeak between the ^{13}C5 retinal resonance and the ^{13}C=O resonance of His211$^{5.46}$ is only consistent with MD simulations where the β-ionone ring is positioned toward the intracellular side of the retinal binding site (Supplementary Table 1).

Supplementary Note 3: Reorientation of the β-ionone ring upon decay of Meta-II.

The crystal structures of Meta-II-opsin[2] or Meta-II-M257Y [15] show that the β-ionone ring of the retinal has the opposite orientation as that observed in rhodopsin and in the Meta-II intermediate trapped at low temperature for NMR (see Supplementary Fig. 1). To probe for retinal ^{13}C18 crosspeaks to any Tyr191^{EL2} carbon, we obtained NMR spectra of rhodopsin and Meta-II containing U-^{13}C tyrosine and regenerated with ^{13}C5-^{13}C18 retinal (Supplementary Fig. 4). As with rhodopsin containing single labeled ^{13}Cζ-tyrosine, no cross peaks to the retinal ^{13}C18 methyl group were observed.

We also assessed whether there is a change in ring orientation during the decay of Meta-II to opsin (Supplementary Fig. 4). For these experiments, the sample was then warmed to 285K for 10 minutes, re-cooled to 200K and the DARR NMR spectrum re-collected. On the basis of the overall intensity loss in the C5-C18 crosspeak, we estimate that there was a loss of ~20% Meta-II after one warm-cool cycle and ~60% after three warm-cool cycles (Supplementary Fig. 4c, black filled circles). The largest increases in tyrosine intensity are associated with a ^{13}C18 methyl resonance that has shifted to ~22.1 ppm. From the 2D plot in Supplementary Fig. 4b, one can see that the tyrosine resonances are associated with a C18 resonance at higher frequency. Supplementary Figure 4e presents rows taken through the diagonal ^{13}C18 methyl resonances at 20.9 ppm and 22.1 ppm. We attribute the C5-C18 crosspeak associated with the 20.9 ppm ^{13}C18 resonance to the original Meta-II intermediate, which is narrower than the 22.1 ppm ^{13}C18 resonance that appears upon Meta-II decay. While the chemical shift of the ^{13}C5 resonance is at 126 ppm in Meta II, the 22.1 ppm resonance exhibits a crosspeak with a ^{13}C5 resonance at 130.9 ppm in samples not labeled with U-^{13}C tyrosine (unpublished results). The 130.9 ppm ^{13}C5 crosspeak overlaps with the crosspeaks between the ^{13}C18 methyl resonance and the ^{13}C-tyrosine resonances corresponding to the Cδ and Cγ carbons (accounting for the larger intensity in this broad resonance as compared to the crosspeak to the Cε carbons at 117 ppm). The high frequency of the ^{13}C5 chemical shift is similar to that in rhodopsin at 131.0 ppm and suggests that the retinal with the flipped ring orientation is bound to Lys296$^{7.43}$ as a protonated Schiff's base. That is, the ^{13}C chemical shifts of the odd numbered carbons of the retinal polyene chain are sensitive to electron delocalization along the chain and are generally higher in frequency (downfield chemical shift) in protonated retinal Schiff bases compared to unprotonated Schiff bases[16].

The observation of the flipped orientation of the retinal in the Meta-II opsin crystal structure may reflect a more open retinal binding site in opsin that allows the β-ionone ring to adopt a lower energy conformation, consistent with the crystallization conditions and with MD simulations showing that the retinal can flip orientation within the opsin binding site[17]. The same situation may exist for the M257Y mutant in which the structural transition to an active state can be achieved by simply adding all-*trans* retinal as an exogenous ligand. We propose that the intermediate we observe in the decay of Meta-II to opsin is Meta-III, which is known to have a protonated Schiff base. Meta-III may form by a change in the conformation of the C=N bond, as found previously[18] or by rotation of the long-axis of the retinal[17]. Both changes would place the Schiff base nitrogen in a position to be protonated by the Glu113$^{3.28}$ carboxyl group. Protonation of Glu113$^{3.28}$ would likely reverse the hydrogen-bonding changes of Glu181^{EL2} and Tyr191^{EL2} observed in the conversion of Meta-I to Meta-II, consistent with the conclusion of Mahalingam

and Vogel [18] that the all-*trans*, 15-*syn* chromophore in Meta-III serves as partial (rather than full) agonist.

Supplementary Note 4. Assignment of the tyrosine-glycine crosspeak in Meta-II.

The assignments of the glycine residues in rhodopsin and Meta-II provide key constraints for the position of the retinal. We have shown that the C20 methyl group has a strong contact with Tyr268$^{6.51}$ (Fig. 1e main text, Supplementary Fig. 2). This methyl group also exhibits a weak crosspeak to a glycine residue, which we previously assigned[19,20] to Gly114$^{3.29}$. Here, we revise that assignment. There are four potential glycine residues near the retinal: Gly114$^{3.29}$, Gly120$^{3.35}$, Gly121$^{3.36}$ and Gly188^{EL2}. We show in the main text that the Gly114$^{3.29}$ and Gly188^{EL2} chemical shifts in Meta-II are at 44.5 and 43.0 ppm, respectively (Fig. 2b). The C20 retinal methyl group exhibits a crosspeak with a glycine at 46.5 ppm, which indicates that the glycine is either Gly120$^{3.35}$ or Gly121$^{3.36}$.

The intensities of the C20-Tyr and C20-Gly crosspeaks relative to the retinal C12-C20 crosspeak reveal that the C20-Gly distance is much longer than the C20-Tyr distance, disagreeing with the distances observed in the Meta-II-opsin crystal structure (Supplementary Fig. 1c). On the basis of the the relative intensity of these crosspeaks, the C20-Gly121$^{3.36}$ distance is estimated to be ~5.5 – 6.0 Å. This distance is longer than the 4.5 – 5 Å separation in the Meta II crystal structures (Supplementary Table 3), but shorter than the 7.3 Å distance predicted on the basis of the MD simulations.

Given the strong intensity of the C20- Tyr268$^{6.51}$ crosspeak, we propose that there is inward tilt of the intracellular end of H6. This motion would serve to shift the position of Tyr268$^{6.51}$. Specifically, it would decrease the distance of the ^{13}Cζ-carbon of tyrosine Tyr268$^{6.51}$ to the retinal ^{13}C20 methyl group, as well as to the ^{13}C14 and ^{13}C15 carbons on the retinal chain, and to increase the distance with retinal ^{13}C10, ^{13}C11 and ^{13}C12 carbons as observed in Fig. 1.

Supplementary Note 5. Interaction of Met288$^{7.35}$ with Tyr191^{EL2}.

The counterion shift mechanism[21] along with chemical shift changes in Ser186^{EL2} (Fig. 3b) argue for a change in the position of the Glu181^{EL2} side chain in the formation of Meta I. The unusual downfield chemical shift of Tyr191^{EL2} in Meta II at 159.3 ppm argues for a strong hydrogen bonding interaction with the Glu181^{EL2} carboxyl group. However, this change does not occur until Meta II (Fig. 3c). To more clearly define the position of Tyr191^{EL2} in Meta II, we

undertook DARR NMR studies on Met-Tyr interactions using rhodopsin labeled with ^{13}Cε-methionine and U-^{13}C tyrosine (Supplementary Fig. 6).

In the rhodopsin crystal structure (PDB ID 1U19), there are five ^{13}Cε-Met - ^{13}Cζ-Tyr pairs that are close in space. However, only three pairs involve tyrosines (^{13}C-Tyr268$^{6.51}$, Tyr191^{EL2} and Tyr192^{EL2}) that would exhibit strong crosspeaks to Met288$^{7.35}$ in a row at 17.2 ppm (Supplementary Fig. 6a). We observe two strong ^{13}Cε-Met-^{13}Cζ-Tyr crosspeaks (assigned to Tyr268$^{6.51}$ at 3.9 Å and Tyr191^{EL2}/Tyr192^{EL2} at 5.2 Å/5.7Å).

Additionally, we observe three ^{13}Cε-Met - ^{13}C=O-Tyr crosspeaks in rhodopsin (Supplementary Fig. 6a). The Tyr268$^{6.51}$ C=O is close to the Met288$^{7.35}$ Cε carbon (4.7 Å). This is the shortest Tyr C=O - Met Cε distance in rhodopsin. Therefore, the most intense ^{13}C=O resonance at ~175 ppm is assigned to Tyr268$^{6.51}$. The Tyr191^{EL2} and Tyr192^{EL2} C=O carbons are more distant from the Met288$^{7.35}$ Cε and generate weaker crosspeaks at 172-173 ppm through spin diffusion.

We lose the ^{13}Cε-Met288$^{7.35}$-^{13}Cζ-Tyr268$^{6.51}$ crosspeak (Supplementary Fig. 6b) as we have assigned the Tyr268$^{6.51}$ resonance in Figs. 1 and 2 at 156.3 ppm. The Tyr191^{EL2} resonance is assigned by its unique ^{13}Cζ resonance at 159.3 ppm [19] which exhibits a strong intra-residue crosspeak to the C=O resonance at 172 ppm. The 172 ppm chemical shift is characteristic of non-helical structure, which would distinguish it from Tyr43$^{1.38}$, Tyr268$^{6.51}$ or other tyrosines within the TM helices. The observation of a strong ^{13}Cε-Met288$^{7.35}$-^{13}C=O-Tyr191^{EL2} crosspeak at 172 ppm in Meta-II is consistent with a shift of Tyr191^{EL2} toward Met288$^{7.35}$. We observe one strong and several weak crosspeaks for ^{13}C=O-labeled tyrosine in Meta II. We assign the strong crosspeak at 172 ppm to Tyr191^{EL2} on the basis of its strong intra-residue crosspeak with the 159.3 ppm Cζ resonance.

The Met288$^{7.35}$ and Ser186^{EL2} chemical shifts are sensitive to their proximity to Glu181^{EL2} and are used to monitor possible movement of Glu181^{EL2}. Glu181^{EL2} motion, along with hydrogen-bonding changes involving Ser186^{EL2}, was previously proposed as part of the counter-ion shift mechanism[21,22]. In the rhodopsin crystal stucture, the Cε-methyl group of Met288$^{7.35}$ is in close proximity to Glu181^{EL2} (3 Å). The decrease in the ^{13}Cε-Met288$^{7.35}$ chemical shift in Meta-II suggests that the distance between Glu181^{EL2} and Met288$^{7.35}$ increases. The Cβ-OH of Ser186^{EL2} is further away from the Glu181^{EL2} side chain carboxyl group (~5 Å). The increase in the ^{13}Cβ Ser186^{EL2} chemical shift in Meta-I suggests that the distance between Glu181^{EL2} and Ser186^{EL2} decreases in the transition to Meta I.

Supplementary Note 6. FTIR analysis of rhodopsin mutants.

FTIR difference spectroscopy provides a complementary approach to NMR for characterizing the changes occurring in individual amino acids between the inactive and active states of rhodopsin. In the difference spectrum, only those vibrations that change during the photoreaction are observed[8]. In Supplementary Fig. 7a, we show for wild-type rhodopsin the difference FTIR spectra between 1620-1850 cm^{-1} for Meta-I (blue) and Meta-IIbH+ (red) minus rhodopsin. These two spectra are the two end-point reference spectra for following the pH titration that shifts the equilibrium between the Meta-I and Meta-II states. Meta-IIbH+ is the Meta II substate in which Glu134$^{3.49}$ has been protonated and the ionic interaction with Arg135$^{3.50}$ is disrupted[23]. There are three signature vibrational bands (Asp83$^{2.50}$, Glu122$^{3.37}$ and amide I) that are analyzed to follow the transition. These bands exhibit characteristic shifts in frequency and intensity between Meta-I and Meta-IIbH+.

Measurements of Meta II formation, helix H6 motion and proton uptake have been used to define a series of Meta-II substates at temperatures above ~4 °C[23]. The first substate, Meta-IIa, corresponds to the deprotonation of the retinal PSB nitrogen and the associated protonation of Glu113$^{3.28}$. This step is followed by UV-vis spectroscopy through the large shift in the retinal λ_{max} upon retinal Schiff base deprotonation. The second substate (Meta-IIb) corresponds to the outward pivoting of helix H6 and can be followed by EPR spectroscopy. The final substate (Meta-IIbH+) involves protonation of Glu134$^{3.49}$.

Supplementary Note 7. Conserved residues involved in the Tyr191^{EL2} trigger.

The location of Tyr268$^{6.51}$ and its role in bridging structural changes involving Glu181^{EL2} and Tyr191^{EL2} in EL2 with Trp265$^{6.48}$ and Phe261$^{6.44}$ have been described in the main text. Much less is known about Tyr191^{EL2} and its interactions with surrounding residues since the sequence of EL2 is not highly conserved outside of specific subfamilies in the family A GPCRs. In this supporting figure, we highlight the cluster of residues surrounding Tyr191^{EL2} that are highly conserved within the visual receptors and influence the lifetime of the Meta-II intermediate and receptor activity.

For example, the residue at position 189 is strictly conserved as a proline in the cone pigments, but as isoleucine in the rod pigments[24]. Ile189^{EL2} is one of two critical residues that distinguish the Meta-II decay rates in the rod and cone pigments and thereby contributes to the sensitivity difference between these two types of visual receptors. Ile189^{EL2}, in turn, interacts with several additional amino acids that are highly conserved in the visual receptor subfamily indicating their importance in terms of structure and/or function.

The picture that emerges is a set of residues that are coupled by packing and hydrogen-bonding interactions whose rearrangement is associated with receptor activation. Several of these residues ($Pro171^{4.60}$, $Trp175^{EL2}$, $Ser176^{EL2}$, $Arg177^{EL2}$) contribute to a cluster in which the removal of hydrogen-bonding interactions leads to receptor unfolding[25]. We propose that retinal isomerization disrupts the packing of the retinal C19 methyl group with $Ile189^{EL2}$ and $Tyr191^{EL2}$, which in turn destabilizes the packing interactions at the extracellular ends of TM helices H5 and H6.

Supplementary References

1. Hornak, V. et al. Light activation of rhodopsin: Insights from molecular dynamics simulations guided by solid-state NMR distance restraints. *J. Mol. Biol.* **396**, 510–527 (2010).

2. Choe, H.W. et al. Crystal structure of metarhodopsin II. *Nature* **471**, 651-655 (2011).

3. Goncalves, J.A., Ahuja, S., Erfani, S., Eilers, M. & Smith, S.O. Structure and function of G protein-coupled receptors using NMR spectroscopy. *Prog. NMR Spectroscopy* **57**, 159-180 (2010).

4. Goncalves, J. et al. Magic angle spinning nuclear magnetic resonance spectroscopy of G protein-coupled receptors. *Methods Enzymol.* **522**, 365-89 (2013).

5. Ahuja, S. et al. 6-s-cis conformation and polar binding pocket of the retinal chromophore in the photoactivated state of rhodopsin. *J. Am. Chem. Soc.* **131**, 15160–15169 (2009).

6. Shriver, J.W., Mateescu, G.D. & Abrahamson, E.W. A proton and carbon-13 nuclear magnetic resonance spectroscopy study of the conformation of a protonated 11-cis-retinal Schiff base. *Biochemistry* **18**, 4785-4792 (1979).

7. Crocker, E. et al. Location of Trp265 in metarhodopsin II: Implications for the activation mechanism of the visual receptor rhodopsin. *J. Mol. Biol.* **357**, 163-172 (2006).

8. Mahalingam, M., Martinez-Mayorga, K., Brown, M.F. & Vogel, R. Two protonation switches control rhodopsin activation in membranes. *Proc. Natl. Acad. Sci. USA* **105**, 17795-17800 (2008).

9. Janz, J.M., Fay, J.F. & Farrens, D.L. Stability of dark state rhodopsin is mediated by a conserved ion pair in intradiscal loop E-2. *J. Biol. Chem.* **278**, 16982-16991 (2003).

10. Madabushi, S. et al. Evolutionary trace of G protein-coupled receptors reveals clusters of residues that determine global and class-specific functions. *J. Biol. Chem.* **279**, 8126-8132 (2004).

11. Kazmin, R. et al. The activation pathway of human rhodopsin in comparison to bovine rhodopsin. *J. Biol. Chem.* **290**, 20117-20127 (2015).

12. Nakamichi, H. & Okada, T. Crystallographic analysis of primary visual photochemistry. *Angew. Chem.-Int. Edit.* **45**, 4270-4273 (2006).

13. Nakamichi, H., Buss, V. & Okada, T. Photoisomerization mechanism of rhodopsin and 9-cis-rhodopsin revealed by X-ray crystallography. *Biophys. J.* **92**, L106-L108 (2007).

14. Leioatts, N. et al. Retinal ligand mobility explains internal hydration and reconciles active rhodopsin structures. *Biochemistry* **53**, 376-385 (2014).

15. Deupi, X. et al. Stabilized G protein binding site in the structure of constitutively active metarhodopsin-II. *Proc. Natl. Acad. Sci. USA* **109**, 119-124 (2012).

16. Harbison, G.S. et al. High-resolution carbon-13 NMR of retinal derivatives in the solid state. *J. Am. Chem. Soc.* **107**, 4809-4816 (1985).

17. Feng, J., Brown, M.F. & Mertz, B. Retinal Flip in Rhodopsin Activation? *Biophys. J.* **108**, 2767-2770 (2015).

18. Mahalingam, M. & Vogel, R. The all-trans-15-syn-retinal chromophore of metarhodopsin III is a partial agonist and not an inverse agonist. *Biochemistry* **45**, 15624-15632 (2006).

19. Ahuja, S. et al. Helix movement is coupled to displacement of the second extracellular loop in rhodopsin activation. *Nat. Struct. Mol. Biol.* **16**, 168-175 (2009).

20. Ahuja, S. et al. Location of the retinal chromophore in the activated state of rhodopsin. *J. Biol. Chem.* **284**, 10190-10201 (2009).

21. Yan, E.C.Y. et al. Retinal counterion switch in the photoactivation of the G protein-coupled receptor rhodopsin. *Proc. Natl. Acad. Sci. USA* **100**, 9262-9267 (2003).

22. Yan, E.C.Y. et al. Photointermediates of the rhodopsin S186A mutant as a probe of the hydrogen-bond network in the chromophore pocket and the mechanism of counterion switch. *J. Phys. Chem. C* **111**, 8843-8848 (2007).

23. Knierim, B., Hofmann, K.P., Ernst, O.P. & Hubbell, W.L. Sequence of late molecular events in the activation of rhodopsin. *Proc. Natl. Acad. Sci. USA* **104**, 20290-20295 (2007).

24. Kuwayama, S., Imai, H., Hirano, T., Terakita, A. & Shichida, Y. Conserved proline residue at position 189 in cone visual pigments as a determinant of molecular properties different from rhodopsins. *Biochemistry* **41**, 15245-15252 (2002).

25. Rader, A.J. et al. Identification of core amino acids stabilizing rhodopsin. *Proc. Natl. Acad. Sci. USA* **101**, 7246-7251 (2004).
